# Optogenetics: Background, Methodological Advances and Potential Applications for Cardiovascular Research and Medicine

**DOI:** 10.3389/fbioe.2019.00466

**Published:** 2020-01-29

**Authors:** Jyotsna Joshi, Michael Rubart, Wuqiang Zhu

**Affiliations:** ^1^Department of Cardiovascular Medicine, Physiology and Biomedical Engineering, Mayo Clinic, Phoenix, AZ, United States; ^2^Department of Pediatrics, Wells Center for Pediatric Research, Indiana University School of Medicine, Indianapolis, IN, United States

**Keywords:** optogenetics, channelrhodopsins, opsin expression, cardiac pacing, arrhythmias, myocardial infarction, cardiac electrophysiology, heterocellular coupling

## Abstract

Optogenetics is an elegant approach of precisely controlling and monitoring the biological functions of a cell, group of cells, tissues, or organs with high temporal and spatial resolution by using optical system and genetic engineering technologies. The field evolved with the need to precisely control neurons and decipher neural circuity and has made great accomplishments in neuroscience. It also evolved in cardiovascular research almost a decade ago and has made considerable progress in both *in vitro* and *in vivo* animal studies. Thus, this review is written with an objective to provide information on the evolution, background, methodical advances, and potential scope of the field for cardiovascular research and medicine. We begin with a review of literatures on optogenetic proteins related to their origin, structure, types, mechanism of action, methods to improve their performance, and the delivery vehicles and methods to express such proteins on target cells and tissues for cardiovascular research. Next, we reviewed historical and recent literatures to demonstrate the scope of optogenetics for cardiovascular research and regenerative medicine and examined that cardiac optogenetics is vital in mimicking heart diseases, understanding the mechanisms of disease progression and also in introducing novel therapies to treat cardiac abnormalities, such as arrhythmias. We also reviewed optogenetics as promising tools in providing high-throughput data for cardiotoxicity screening in drug development and also in deciphering dynamic roles of signaling moieties in cell signaling. Finally, we put forth considerations on the need of scaling up of the optogenetic system, clinically relevant *in vivo* and *in silico* models, light attenuation issues, and concerns over the level, immune reactions, toxicity, and ectopic expression with opsin expression. Detailed investigations on such considerations would accelerate the translation of cardiac optogenetics from present *in vitro* and *in vivo* animal studies to clinical therapies.

## Introduction

The term “optogenetics” was first introduced in 2006 by Deisseroth et al. and it broadly refers to an elegant approach that utilizes genetic engineering and optical technology to control and monitor biological functions of isolated or *in situ* cells, tissues, organs or organisms, modified to express photosensitive proteins (Deisseroth et al., 2006; Miesenböck, 2009; Entcheva, 2013; Jiang et al., 2017; Koopman et al., 2017). The photosensitive proteins are optical sensors or optical actuators which provide fluorescent readout for changes in biological activities or allow light to manipulate the cellular biological functions, respectively (Shui et al., 2014; Jiang et al., 2017). Photosensitization of a specific cell type, tissue, or organ of interest together with the application of defined light stimulation and efficient light detection systems has enabled optogenetics to perturb and monitor biological functions non-invasively with high spatiotemporal resolution (Boyden et al., 2005; Park et al., 2014; Koopman et al., 2017). Biomedical applications of optogenetics have evolved from neuroscience with needs to precisely and rapidly control individual cells in a vertebrate brain for deciphering the neural circuitries underlying behavior and diseases, replacing approaches which were not precise enough to target specific neuron populations, were highly invasive, or were too slow in kinetics (Boyden et al., 2005; Zhang et al., 2010; Mei and Zhang, 2012; Guru et al., 2015). Historically, the concept of optogenetics was conceived for neuroscience in 1979 with the suggestion from Francis Crick on the potential utility of light in providing rapid spatiotemporal control for targeting specific neurons; however, during that time neuroscientists did not know methods to apply such photosensitive proteins in neuroscience (Deisseroth, 2011). Nevertheless, microbiologists had already known during that time on the existence of photosensitive proteins which regulates ion flow across the plasma membrane in some microorganisms (Oesterhelt and Stoeckenius, 1971; Matsuno-Yagi and Mukohata, 1977; Deisseroth, 2011). The seminal development in the field evolved with a pioneering study by Nagel et al. (2003) which demonstrated the feasibility to express microbial opsins, a light-sensitive ion channel protein, in non-excitable mammalian cells and enable fast, light-induced cell depolarization by tens of mV. Similarly, another pioneering study by Boyden et al. (2005) demonstrated the efficacy of light in modulating the electrical excitability of neurons with high spatial and temporal resolution upon expression of microbial opsins in mammalian neurons. These studies led to an unprecedented growth of optogenetics in different areas of neuroscience but the field was largely unexplored for cardiovascular research at that time (Deisseroth, 2011; Entcheva, 2013; Guru et al., 2015). Fortunately, a new realm of cardiac optogenetics was laid by Bruegmann et al. and Arrenberg et al., after their remarkable studies on the successful applications of optogenetics for controlling cardiomyocyte excitability both *in vitro* and *in vivo* of adult mouse hearts and on the localization of pacemaker cells in the developing zebrafish heart, respectively (Arrenberg et al., 2010; Bruegmann et al., 2010; Entcheva, 2013).

In this review, we will begin with background on the photosensitive proteins commonly used in optogenetics, including their origin, chemical composition, structures, types, and biophysical properties. Then, we will review common genetic engineering approaches for expression of optogenetic proteins in the target cells, tissues and organs. Finally, we will focus on the potential applications of optogenetics for cardiovascular research and medicine and will conclude with some considerations for the translation of the field for clinical therapies.

## Optogenetic Proteins

### Background

All organisms from archaebacteria to humans express photoreceptor proteins, called rhodopsins, which provide them the unique ability to “sense and respond” to light (Kato et al., 2012; Ernst et al., 2013). However, based on the primary sequence and mode of action, opsins are categorized as microbial (type I) opsins and animal (type II) opsins; the former type is found in microbes, such as archaea, eubacteria, fungi, and algae, and the latter is found in animals and humans (Kato et al., 2012). Microbial opsins include light-driven ion pumps, such as bacteriorhodopsins (BRs) and halorhodopsins (HRs), ion channels, such as channelrhodopsins (ChRs), and sensors, such as sensory rhodopsin I and II (SRI and SRII) (Govorunova et al., 2017). Upon light stimulation, these opsins mediate transmembrane ionic currents and elicit specific biological responses, such as phototaxis and photophobia, by coupling with specific transducers (Nagel et al., 2003; Ernst et al., 2013). Animal opsins indirectly mediate transmembrane ionic potential by coupling to G-protein-mediated transduction pathways and are primarily involved in dim light vision and circadian clock (Kato et al., 2012; Ernst et al., 2013). Microbial opsins are commonly used in optogenetic research because of their faster kinetics and relative ease of genetic engineering of a single component protein, compared to animal opsins (Guru et al., 2015).

### Structure and Basic Mechanism

Microbial and animal rhodopsins share architectural similarities and both are composed of seven transmembrane α-helices, called opsins (also termed as apoproteins) and a photosensitive chromophore, called retinal (Govorunova et al., 2017). Opsins are designated as helix A to G in type I and as TM 1 to 7 in type II, with their N-terminus on the outside of the cell and their C-terminus on the inside (Ernst et al., 2013). On the other hand, retinal, an aldehyde of vitamin A and derivative of β-carotene, is covalently linked to ε-amino group of lysine side chain on helix G or TM7 via retinal Schiff base (RSB) linkage (Kato et al., 2012; Ernst et al., 2013). Upon illumination at a specific wavelength, retinal in microbial rhodopsins usually undergoes isomerization from all-trans to 13-cis that initiates a series of cyclic reactions, known as photocycle, unique for each microbial rhodopsin type (Bayraktar et al., 2012; Ernst et al., 2013). This causes generation of unique photo-intermediates that induces conformational change of proteins, which causes channel opening, closing and ionic conductance of the rhodopsin (Bayraktar et al., 2012; Ernst et al., 2013).

### Channelrhodopsin

**Figure 1:**
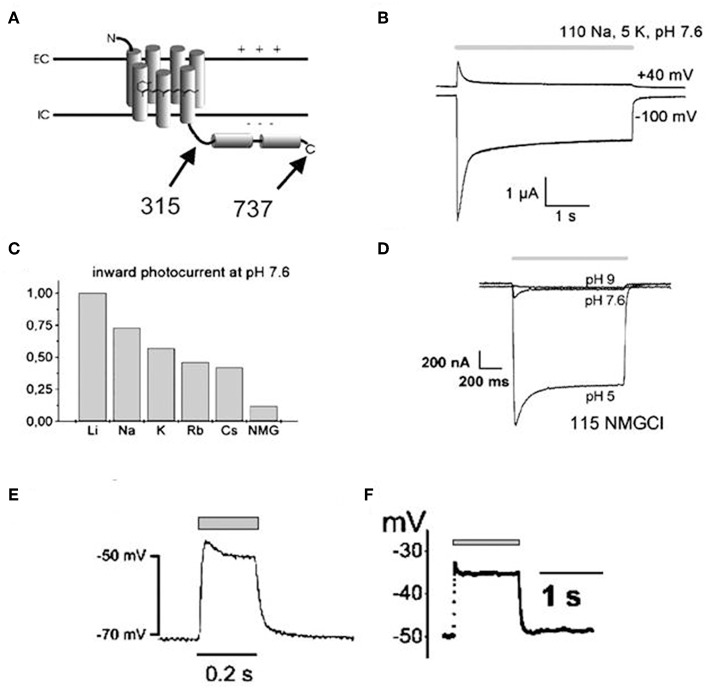
Illustrations showing structural topology of Channelopsin-2 (Chop2) and the dependency of light-induced currents of Channelrhodopsin (ChR2) on both cellular microenvironment and illumination parameters. Both variants of Chop2, full length Chop2-737 and truncated Chop2-315 **(A)**, elicited large photocurrents upon light stimulation when expressed in oocytes. The channel desensitizes during continuous blue light illumination (450 ± 25 nm), evidenced by the decay of the photocurrent to a steady-state level **(B)**, magnitude and direction of the photocurrent varies with the membrane potential **(B)**, magnitude of photocurrents also differs when kept in solution of different cations **(C)**, magnitude of photocurrent also depends on the extracellular pH **(D)**, and depolarization of cell membrane (millivolt) can be achieved rapidly upon photosensitization with blue light illumination both in oocytes **(E)** and HEK293 cells **(F)**. Reproduced with permission from Nagel et al. (2003) and Chang Liao et al. (2015).

Channelrhodopsin (ChR) was first discovered in unicellular green algae *Chlamydomonas reinhardtii* where it provides the algal eyespot the ability to sense and respond to light (Nagel et al., 2002, 2003; Ernst et al., 2013). Thus, following light stimulation, the primary depolarization in the plasma membrane of the algal eyespot is transferred to the flagellar membrane, which causes reorientation of algae toward light source and induces phototaxis and photophobic responses (Ernst et al., 2013). The green algae encode two forms of ChR, ChR1 and ChR2, both are involved in light-mediated responses but their scope and knowledge of mechanism of action were very limited until their cloning by Nagel et al. in the *Xenopus* oocytes and mammalian cells (Nagel et al., 2002, 2003). In 2003, Nagel et al. first cloned channelopsin-2 (Chop2) in Xenopus oocytes, in the presence of all-trans retinal, to determine if functional rhodopsin (having covalently linked retinal, ChR2) can be obtained and also to examine their functional characteristics (Nagel et al., 2003). They noted that magnitude, direction and functional characteristics of light-induced photocurrents from expressed Channelrhodopsin (ChR2) are guided by both the cellular microenvironment and the properties of blue light stimulus, such as intensity and duration (Figures 1A–F; Nagel et al., 2003). ChR1 and ChR2 are both common in optogenetics; however, the latter is getting more attention since heterologous channelopsin-2 (Chop2) exhibit capability to link with an endogenous all-trans-retinal and form functional ChR2 in the mammalian system (Lin et al., 2009). In addition, ChR2 exhibits fast onset kinetics (milliseconds), show capability to reliably produce action potentials from high frequency light pulses (milliseconds), and forms functional complexes in most vertebrate systems without the need for exogenous supplementation (due to the adequate availability of all-trans-retinal); thus, ChR2 has remained as a prototypical and very commonly used optogenetic tool (Mei and Zhang, 2012; Entcheva, 2013).

### Halorhodopsin

Halorhodopsin (HR) is found in halobacteria where it is utilized for light-induced, inward-chloride transport across the outer membrane (Lanyi, 1986). Thus, in physiological transmembrane Cl^−^ gradients, HR from an archaeon, *Natronomonas pharaonis* (NpHR), is one of the commonly used optogenetic proteins for generating hyperpolarizing, i.e., inhibitory currents in mammalian cells (Guru et al., 2015; Wiegert et al., 2017). In this regard, Han and Boyden (2007) demonstrated the feasibility of NpHR to rapidly and reversibly silence targeted neurons using yellow light pulses (560 ± 27.5 nm, 10 mW/mm^2^). They observed that hippocampal neurons, expressing NpHR, elicited outward currents with fast kinetics (<20 ms) when voltage-clamped and produced rapid membrane hyperpolarization (~100 ms) when current-clamped (Han and Boyden, 2007). Thus, their study showed that upon heterologous expression of NpHR in targeted regions and upon application of defined light stimulus, specific population of neurons can be reversibly and reliably silenced with high spatiotemporal resolution (Han and Boyden, 2007). Since then several investigators have explored mechanisms to generate enhanced versions of NpHR pumps (eNpHR) for safe and improved membrane trafficking and to further improve the ability to render mammalian cells electrically inexcitable (Gradinaru et al., 2008; Guru et al., 2015). In the context of cardiac optogenetics, Arrenberg et al. (2010) first reported the use of eNpHR in zebrafish hearts to cause hyperpolarization of myocardial cells and stop heart contractions. Since then several investigators have reported the use of eNpHR and other inhibitory optogenetic tools, such as achaerhodopsin (Arch), for inhibition of cardiomyocytes activity (Abilez, 2012; Nussinovitch et al., 2014; Park et al., 2014).

### Optogenetic Sensors

Multiple optogenetic sensors are available to monitor changes in electrical and biochemical parameters, such as membrane voltage, calcium and chloride concentrations, pH, neurotransmitter release, etc. (Tan et al., 2015; Jiang et al., 2017). Structurally, these sensors are composed of a sensing domain linked to a single or a pair of fluorescent proteins (Koopman et al., 2017). Thus, when voltage, pH, calcium, or any other physiological parameter changes, the conformation of the sensing domain changes which in turn influences the optical properties of the linked fluorescent proteins, such as changes in their brightness or Förster resonance energy transfer (FRET) (Koopman et al., 2017). Genetically encoded calcium indicators (GECIs) have made great progress and are available with a wide spectral sensitivity, binding affinity and sub-cellular localization capability which have allowed subtle monitoring of calcium changes both in *in vivo* and *in situ* settings, not attainable with conventional optical dyes (Shui et al., 2014; Koopman et al., 2017). Historically, Nakai et al. (2001) first developed GECI, named GCaMP, by connecting N-terminus of circularly permutated enhanced GFP (cpEGFP) to the M13 fragment of myosin light chain kinase and by connecting the C terminus of cpEGFP to calmodulin (CaM); thus, when calcium binds to CaM, the conformation of cpEGFP changes altering the fluorescence intensity and thereby allowing sensing of calcium changes. Since then, GCaMP has remained as a popular calcium sensor in the field; nevertheless, several GCaMP variants are being engineered to account for issues with nuclear accumulation, toxicities and dysregulated calcium signaling and also to provide enhanced calcium sensing capacity (Nakai et al., 2001; Yang et al., 2018).

Next, genetically encoded voltage indicators (GEVIs), based on bacterial rhodopsin or voltage sensing domain (VSD) of voltage sensitive proteins, are also gaining greater attention in the field (Koopman et al., 2017). VSD-based sensors evolved with the discovery of VSD linked to phosphatase in the sea squirt *Ciona intestinalis* (CiVSP) and with the first construction and efficient plasma membrane localization of the voltage-sensitive fluorescent probe (VSFP2.1) in mammalian cells by Dimitrov et al. (Murata et al., 2005; Dimitrov et al., 2007; Lundby et al., 2008; Akemann et al., 2009). The probe was made by fusing VSD of *C. intestinalis* to a fluorescent reporter and further through mutations to cause sensor activation within the physiological range of the neuronal membrane potential (Dimitrov et al., 2007; Lundby et al., 2008). Structurally, the VSD of CiVSP contains four transmembrane helices (S1–S4) linked to phosphatase while VSFP2.1 and its further engineered version VSFP2.3 (formed by eliminating specific amino acids) are linked to fluorescent reporter proteins (such as cyan and yellow fluorescent proteins) to indicate membrane potential changes through FRET (Dimitrov et al., 2007; Lundby et al., 2008; Akemann et al., 2009). In this aspect, both VSFP2.1 and VSFP2.3 exhibit excellent membrane targeting ability; however, improved versions of VSFP are also being investigated specifically for neuroscience application for their slower fluorescence readout [e.g., time constant (>100 ms) for fluorescence activation at −10 mV for VSFP2.3; (Lundby et al., 2008)].

### Strategies to Improve Opsin Performance

Several opsin variants have evolved through targeted mutations and chimera creation to meet specific optical and biological requirements, such as spectral properties, photosensitivity, photocurrents, kinetics, specific membrane trafficking capacity, and temporal resolution (Ernst et al., 2013; Guru et al., 2015). For instance, ultrafast opsins are evolving to provide faster kinetics, step-function opsins are useful in extending the time of channel open state while red shifted opsins are promising for non-invasive and deeper tissue stimulation as red light exhibits reduced scattering and absorption (Lin et al., 2013; Klapoetke et al., 2014; Guru et al., 2015). Specifically, several ChR variants have also evolved to address concerns of fast inactivation with ChR2 (i.e., decline in the response upon repeated light stimulation) and insufficient depolarization with ChR1 (i.e., limitation of the number of protons permeating through the channel upon illumination; Lin et al., 2009). Lin et al. (2009) first engineered ChR variants artificially by making chimeras of channel opsins of ChR1 (Chop1) and ChR2 (Chop2) and by further mutating the residues around retinal-binding pockets. They found that: (i) the resulting chimera ChEF, with crossover site at loop E-F, retained reduced inactivation of ChR1 but allowed additional permeation of sodium and potassium ions, (ii) ChIEF, a CHEF variant where isoleucine 170 is mutated to valine, showed improved kinetics and, (iii) both these variants exhibited membrane depolarization with higher temporal precision when compared to ChR2 and produced consistent photo response upon illumination at higher frequencies (>25 and 50 Hz for ChEF and ChIEF, respectively; Lin et al., 2009).

Thus, several ChR2 variants have evolved to meet different requirements, such as higher conductivity (ChR2-H134R), higher calcium permeability and light sensitivity (CatCh), increased expression and photocurrent (ChR2-XXL), and higher tissue penetrability (ReaChR) (Boyle et al., 2018). For instance, several red-shifted ChR variants have been engineered since ChR2 typically show excitation maximum at 460 nm (Guru et al., 2015). The first identified red-shifted opsin variant VChR1, discovered in *Volvox carteri*, shows maximum excitation at 535 nm; however, it is of limited utility due to its reduced membrane trafficking and low photosensitivity in mammalian systems (Lin et al., 2013; Guru et al., 2015). To address such limitations, Lin et al. (2013) engineered red-activatable channel rhodopsin (ReaChR) by using VChR1 as a template and making several substitutions and mutations in the protein domain to obtain improved red-shifted opsin variant with enhanced membrane trafficking, faster kinetics, improved photosensitivity, and a significantly higher red-shift (maximum response ~590–630 nm excitation). Interestingly, they demonstrated that ReaChR enables optical control of neural spiking in brains of mice with intact skulls (Lin et al., 2013). On the contrary, Nyns et al. used 470 nm light pulse for ReaChR-mediated depolarization and pacing in rat hearts, which contradicts the earlier studies on the red-shifted response of ReaChR (Nyns et al., 2016).

Similarly, new variants of GCaMP are evolving via mutations to improve signal and membrane trafficking since conventional GCaMP are hindered by their tendency to accumulate in the nuclei, produce dysregulated calcium dynamics and cause cell damage and toxicity (Shui et al., 2014; Yang et al., 2018). In this regard, Yang et al. (2018) reported that GCaMP containing CaM interferes with the L-type calcium channel that introduces disruption in calcium signaling and gene expression. Then, they engineered GCaMP-X, a GCaMP variant having additional apoCaM-binding protection motif and an extra tag for specific subcellular localization in the conventional GCaMP, and demonstrated the robustness of the new variant which does not disrupt calcium signaling (Yang et al., 2018). Similarly, several variants of voltage sensors are evolving to improve the kinetics, sensitivity, and reduce the signal artifacts when compared to the results of the conventional voltage sensors (Mei and Zhang, 2012; Xu et al., 2017). GEVIs, based on VSD of *C. intestinalis*, have evolved in various versions, such as from conventional conjugation with FRET pairs (e.g., VSFP2.1and VSFP 2.3 for nullifying motion and blood flow artifacts) to conjugation with single fluorescent protein (for overcoming the broad spectrum barriers associated with FRET system) and now further to mutations in the fluorescent proteins and linker optimization (e.g., VSFP3.1 for enhancing the sensitivity; Xu et al., 2017). A summary on the optogenetic proteins, their source, functions and potential applications in cardiovascular research and medicine is provided in Table 1.

**Table 1:** A list of optogenetic proteins in cardiovascular research and medicine.

**Optogenetic proteins**	**Sources**	**Functions and applications**
Channelrhodopsin (ChR)	Unicellular algae *Chlamydomonas reinhardtii*	Light gated cation-selective membrane channel, provides algal eye ability to sense light; Depolarizing tool in optogenetics
Halorhodopsin (HR)	Halobacteria	Induces light-gated inward directed chloride transport; Inhibitory tool in optogenetics
GCaMP	Calmodulin-based	Calcium sensor
VSFP 2.1	Derived from voltage Sensing domain of sea Squirt *Ciona intestinalis*	Indicator of membrane potential
VChR1	Multicellular algae *Volvox corteri*	Red-shifted opsin variant; Maximal excitation at 535 nm
G-protein modulating opsins	Natural and engineered	Modulates cell signaling
JellyOp	Jelly fish	Gs-protein coupled photoreceptor
Melanopsin	Ganglion Cells of the retina	Gq-coupled photoreceptor; Regulation of circadian rhythm

## Opsin Expression

### Background

Various delivery methods are being explored to express optogenetic proteins in cardiac cells and tissues. Such methods may employ direct delivery of genes (with or without vehicles) and utilize physical cues, such as electric field (electroporation), ultrasound waves (sonoporation), laser pulse (photoporation), hydrodynamic pressure (hydroporation) etc., to enhance gene delivery (Ramamoorth and Narvekar, 2015). Newer methods are also investigating indirect photosensitization method via transplantation of photosensitive cells in the target regions (Gruber et al., 2018). A gene delivery method is ideal for cardiac optogenetics if it is robust, efficient, stable, scalable, and also provides sufficient packaging capacity but elicits minimal off-target, inflammation, disease, toxicity, and mutagenesis (Wasala et al., 2011; Lentz et al., 2012). Physical cues-mediated gene delivery does not evoke concerns of mutations, immune, or inflammatory reactions but poses threats on cell viability since such methods cause irreversible pores in cell membrane (Ramamoorth and Narvekar, 2015). Next, non-viral vehicles, such as nanoparticles and dendrimers, also offer safe, easy and cost-effective means of delivering genes but are hindered by low delivery efficiency and low transgene expression (Ramamoorth and Narvekar, 2015). On the other hand, viruses possess extraordinary ability to invade living cells and organisms and are thus widely used in gene delivery (Wasala et al., 2011). Nevertheless, viruses are prone to cause disease, inflammation, toxicity, and mutagenesis when used during gene delivery. Thus newer strategies are focused to utilize viral invasion ability but limit viral concerns by segregating viral components into multiple constructs (Vannucci et al., 2013). Other strategies, such as “tandem-cell unit” (TCU), are investigating the utility of *in vivo* delivery of donor cells, such as stem cells or non-excitable cells expressing optogenetic proteins, for electrical coupling to the native cardiomyocytes and allowing optical control of the cardiomyocytes or intact heart using exogenously supplied photosensitive cells (Jia et al., 2011).

### Plasmid-Mediated Opsin Expression

Plasmids are extrachromosomal circular DNA molecules (sizes <1 kbp to >200 kbp) and possess ability to replicate independent of the host genome (Cooper et al., 2000; Williams and Kingston, 2011). Structurally, they are composed of: (1) a gene for antibiotic resistance regulated by a promoter, (2) an origin of replication which supports plasmid replication and survival, and (3) cloning sites flanked by restriction endonuclease sites, which provide sites for insertion of transgene constructs (Cooper et al., 2000; Williams and Kingston, 2011; Hardee et al., 2017).

They are very common vectors for molecular cloning in clinical trials because of their safety, negligible risk of oncogenesis, reduced immunogenicity, and adequate packaging capacity (Cooper et al., 2000; Williams and Kingston, 2011; Hardee et al., 2017). Historically, Lin et al. first demonstrated the ability of the heart to intake and express naked plasmid DNA following direct myocardial injection; since then plasmid has remained as a simple and common vector for gene delivery in heart (Lin et al., 1990; Wasala et al., 2011). However, plasmid vectors are mainly hindered by their low transduction efficiency owing to their large size (Williams and Kingston, 2011; Hardee et al., 2017). Thus, several attempts are being explored to improve their efficiency, such as: (1) length shortening: to improve transduction efficiency, (2) physical cues: to enhance penetration into the target cells, (3) cationic vehicles: to protect from getting digested by nucleases and to enhance cellular uptake, (4) modifications in the transgene promoter and use of nuclear localization signals: to improve target-specific transduction efficiency, etc. (Wasala et al., 2011; Williams and Kingston, 2011; Ramamoorth and Narvekar, 2015; Hardee et al., 2017). Nonetheless, these methods may render plasmid susceptible to strand breakage or genomic integration, reducing their therapeutic efficiency (Hardee et al., 2017), or may pose safety concerns due to high voltage requirements with electroporation (Williams and Kingston, 2011). Hence, plasmids are widely engineered to carry viral components for efficient opsin expression, examples of which are detailed in the subsequent sections.

### Viral Vector-Mediated Opsin Expression

#### Adenovirus

Adenoviruses host linear double-stranded DNA, with ~30-kb of the genome, and measure ~70–100 nm (Wasala et al., 2011; Lentz et al., 2012). Applications of recombinant adenovirus for cardiac gene delivery started in the 1990s and have been applied in animal studies and humans (Wasala et al., 2011). These viruses are more efficient than plasmids for cardiac gene therapy and do not integrate with the host genome (Wasala et al., 2011; Lentz et al., 2012). However, prime concerns impeding the clinical success of these vectors include toxicity, immunogenicity and unstable gene expression (Wasala et al., 2011; Lentz et al., 2012).

#### Lentivirus

##### Background

From the last two decades, lentiviruses have been gaining great attention for gene delivery in pre-clinical as well as in clinical studies (Gandara et al., 2018). They are gaining wide popularity for their well-defined characterization, safer new generations, ability to effectively deliver genetic information in both mitotic and non-mitotic cells, and provide long-term and high transgene expression (Wasala et al., 2011; Gandara et al., 2018). They measure ~100 nm and consist of two single-stranded RNA molecules, which undergo reverse transcription and integrate into the host genome during their life cycle (Lentz et al., 2012; Milone and O'doherty, 2018). Thus, gene delivery systems using lentivirus minimize the risks associated with viral particle formation and oncogenesis, but render their machinery to effectively transduce and express target genes (Vannucci et al., 2013; Gandara et al., 2018). One of such milestones is the introduction of lenti-vector system designed to reduce viral genomic load and segregate viral genome in multiple constructs, namely vector construct, packaging construct and envelop construct, so that chances of recombination, viral particle formation, oncogenesis, and mutagenesis are minimized or avoided (Durand and Cimarelli, 2011; Vannucci et al., 2013).

##### Lentiviral vector

The first generation of lentiviral system consists of three constructs each in three plasmids, where a packaging construct contains whole HIV genome except env, an envelope construct encodes env, and a vector construct is flanked by two WT long terminal repeats and it contains the transgene, with all viral components removed except the encapsidation signal (psi) and Rev recognition element (RRE) required for the export of vector and transfer of un-spliced mRNA from nucleus to cytoplasm (Durand and Cimarelli, 2011; Vannucci et al., 2013). In the second generation, the vector is minimally modified while the packaging construct is devoid of accessory genes (such as vif, vpr, vpu, and nef that add no advantage for vector production) but includes Tat and Rev; Tat is required for LTR-driven transcription and Rev is needed for mRNA transport (Dull et al., 1998; Durand and Cimarelli, 2011; Milone and O'doherty, 2018). In the third generation, the vector is made replication-incompetent and self-inactivating (SIN) type by deleting U3 region of 5′LTR (to prevent promoter activity of 5′LTR) and replacing it with an eukaryotic promoter (EP) (to drive the transgene expression) and eliminating Tat from the packaging construct but providing a fourth plasmid for Rev (Durand and Cimarelli, 2011; Vannucci et al., 2013; Gandara et al., 2018; Milone and O'doherty, 2018). In addition, the third generation vector allows incorporation of additional short non-coding sequence, poly purine central tract (cPPT), and regulatory element, a woodchuck hepatitis post-transcriptional regulatory element (W) for enhancing encapsidation efficiency and improving post-transcriptional processing of transgene RNA, respectively (Howarth et al., 2010; Vannucci et al., 2013). Additionally, their ability to accommodate heterologous envelop proteins “env” (pseudotyping) further enhances their tissue tropism and transduction efficiency (Durand and Cimarelli, 2011).

##### Opsin expression method using lentiviral vectors for cardiovascular research

**Figure 2:**
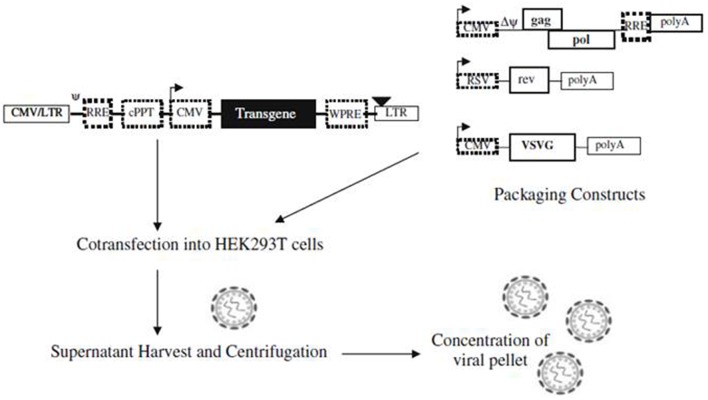
A schematic illustration showing production of lentiviral vectors. Lentiviral vectors are produced via co-transfection of packaging constructs and shuttle vector, containing the transgene, on packaging cell line (e.g., HEK293T). Viral particles are formed upon co-transfection and released into the supernatant via budding process, which can be concentrated before transduction into target cells. Reproduced with permission from Howarth et al. (2010).

Abilez (2012) demonstrated the ability to optically stimulate and inhibit human induced pluripotent stem cell-derived cardiomyocytes (iPSC-CM) colonies using second generation lentiviral system. The tested lentiviral vectors were pLenti-EF1α-ChR2-eYFP-WPRE(pLECYT) and pLenti-EF1α-NpHR1.0-mCherry-WPRE(pLENMT), which were enveloped in lentiviral plasmids (pLenti) and consist of lentiviral constructs composed of human elongation factor 1α (EF1α) promoter, ChR2 or NpHR1.0 transgene, YFP or mCherry fluorescent probes, and WPRE post transcriptional regulatory element (Abilez, 2012). Abilez first produced high titer of lentivirus by co-infecting 293FT (packaging cells) with pLECYT, pLENMT, pCMVRΔ8.74 (containing gag and pol), pMD2.G (containing vesicular stomatitis virus G protein VSVg, a pantropic envelope Durand and Cimarelli, 2011, and calcium phosphate (for transfection) (Abilez, 2012; Zacchigna et al., 2014). Such process would cause release of viral particles in the supernatant via the budding process from the packaging cells and the particles can be collected and centrifuged for further concentration (Figure 2; Howarth et al., 2010). Accordingly, Abilez transduced hiPSCs with such viral particles, allowed transduced cells to differentiate into cardiomyocytes and optically stimulated at days 15–30 of differentiation. It was reported that transduced colonies of hiPSC-CM were optically controlled, showing synchronous stimulation and inhibition upon appropriate light stimulation (Abilez, 2012).

#### Adeno-Associated Virus

##### Background

AAV has emerged as one of the most promising gene delivery systems for both pre-clinical and clinical gene therapies, exceeding 170 clinical trials with excellent clinical safety data (Wasala et al., 2011; Schnoedt and Buening, 2017). Both lentivirus and AAV offer stable long-term and high expression level, but the latter lowers mutagenesis concerns as they do not integrate into the host genome (Lentz et al., 2012; Mei and Zhang, 2012) and is non-pathogenic as it requires helper virus for efficient replication (Schnoedt and Buening, 2017).

##### AAV vector

Structurally, AAV measure ~18–25 nm and consist of a single stranded DNA genome, spanning ~4.7 kb pairs. The viral genome consists of three open reading frames, rep and cap regions codes for replication-related proteins and viral capsid proteins (VP1, VP2, and VP3), respectively while AAP region encodes protein for virion assembly (Zacchigna et al., 2014). The viral genome is flanked by two ITR, each of 145-bp, and are the only required elements for packaging genome into the capsid (Schultz and Chamberlain, 2008; Zacchigna et al., 2014). Thus, in a recombinant AAV (rAAV) vector, viral coding region is replaced by transgene of interest (up to 5 kb) with intact ITRs (Schultz and Chamberlain, 2008; Zacchigna et al., 2014). In specific, rAAV vectors are produced by segregating the genomic content between two plasmids, where AAV vector plasmid will carry the transgene, flanked by ITRs, while a helper plasmid will encode AAV capsid proteins (Howarth et al., 2010). AAV serotype is categorized based on the amino acid sequence of the capsid proteins, where AAV-9 serotype is reported to exhibit robust cardiac tissue tropism compared to other serotypes (Wasala et al., 2011; Lentz et al., 2012). However, the cardiac transduction efficiency of these serotypes is additionally governed by route of gene delivery, presence or absence of helper viral genes and cell cycle status (mitotic vs. non-mitotic; Zacchigna et al., 2014).

##### Opsin expression method using AAV vectors for cardiovascular research

Vogt et al. (2015) utilized AAV vector AAV9-CAG-hChR2(H134R)-mCherry (Penn Vector Core, University of Pennsylvania) to deliver ChR2 gene in the hearts of wild-type mice. They selected AAV9 serotype, ubiquitous promoter CAG (chicken-β-actin promoter) and humanized and mutated ChR2 i.e., hChR2(H134R) in their vector to provide greater tropism to cardiomyocytes, higher transduction efficiency, and larger photocurrents, respectively, while m-Cherry was selected as fluorescent probe (Nussinovitch and Gepstein, 2015; Vogt et al., 2015). For transduction, they first diluted ~2^*^10^11^ genome copies of AAV9-CAG-hChR2(H134R)-mCherry in 100 μl of PBS and injected intravenously (left jugular vein) in anesthetized CD1 wild-type mice (Vogt et al., 2015). They detected that mCherry fluoresces ~ 4–10 weeks in the whole mice hearts and, upon dissociation, more than 50% of cardiomyocytes were observed to be mCherry-positive. Interestingly, action potentials were evoked upon stimulation with short pulses of blue light (1 ms, 5 mW mm^−2^) in the dissociated transduced cardiomyocytes. They also reported that ventricular stimulations were evoked on the transduced mice hearts by optical pacing (shown by broad QRS complexes) and the responses were dependent on the illumination parameters, such as light intensity and irradiation area. These results depict the ability of AAV vector in successful transduction of the optogenetic proteins in the intact cardiomyocytes (Vogt et al., 2015).

### Strategies to Enhance and Targeted Transgene Expression

Structurally, transgenes should include promoters, introns, protein coding sequence, and poly(A) site for successful gene expression, where (1) promoters provide transcription start site and transcription regulatory sequence to control transgene expression both temporally and spatially, (2) introns play a role in mRNA stabilization and translocation from nucleus to the cytoplasm, (3) protein coding sequence enables ribosome scan and recognize start and stop sites for mRNA, and (4) poly(A) site forms the transcriptional stop signals (Brinster et al., 1988; Huang and Gorman, 1990; Haruyama et al., 2009). Cell-specific promoters provide restricted expression of the transgene to the specific cell types or tissue regions of interest (Mei and Zhang, 2012). However, some cell-specific promoters possess long regulatory elements which may not be applicable with some gene delivery methods with limited packaging length, such as AAV and lentivirus (~2 kb for AAV and ~5 kb for lentivirus; Mei and Zhang, 2012; Guru et al., 2015). To eliminate such shortcomings, strong ubiquitous promoters are utilized for high opsin expression but the expression is made site-specific by using conditional expression systems, such as Cre recombinase (Mei and Zhang, 2012; Guru et al., 2015). Thus, when viral vectors containing “floxed-stop” or “floxed-inverse” cassettes are delivered, only the targeted cells, expressing the Cre recombinase, will correct the orientation and allow expression of the targeted gene, which enables cell-specific high expression (Madisen et al., 2012; Guru et al., 2015). For instance, Zaglia et al. (2015) selectively expressed ChR2 either on working cardiomyocytes or on conducting cardiomyocytes by breeding transgenic mice expressing double-floxed-ChR2 with strains expressing Cre recombinase using promoter either for working cardiomyocytes (MyHC-CRE) or conducting myocytes (Cx40-Cre), respectively.

Next, dual vector strategy is being researched to address concerns of small packaging limit for transgene expression which allows single vector genome to be divided into two independent virions that subsequently gets reconstituted (Wasala et al., 2011; Mei and Zhang, 2012). Another concern is the rate limiting second strand synthesis of ssDNA with AAV which confers them relatively low transduction efficiency in the absence of helper viral genes (Ferrari et al., 1996; Schultz and Chamberlain, 2008). Self-complementary (scAAV) vectors are being investigated to eliminate the dependence on rate-limiting second strand synthesis step, where these vectors contain centrally located mutated ITR and two copies of transgene sequences, inverted and complementary to each that allow genome to self-anneal (Lentz et al., 2012; Zacchigna et al., 2014). In addition, approaches on the efficient and safe *in vivo* gene delivery strategies, optimal dose requirements, and modifications on the vector design and vector variants for higher tropism and resistance to neutralizing antibodies etc. are being explored to enhance transgene expression for *in vivo* and clinical applications (Chamberlain et al., 2017; Ishikawa et al., 2018).

## Potential Applications of Optogenetics for Cardiovascular Medicine

### Cardiac Conduction System

Arrenberg et al. (2010) demonstrated that optical interrogation of ChR2- and NpHR- expressing hearts of developing zebrafish will provide a mechanism to map the time-dependent development and convergence of cardiac conduction system. They illuminated zebra fish hearts, from embryonic to larval stage (1–5 dpf), over different heart regions with varying illumination surfaces and light intensities in order to locate the distribution/ convergence and the nature of the pacemaker cells of the conduction system during their development. Illumination of larger area of venous pole at 1 dpf stopped beating of whole heart while illumination of right part of the sinoatrial ring stopped beating at 2 dpf, since illumination would inhibit NpHR expressing pacemaker cells and cause the heart to stop beating (Arrenberg et al., 2010). Similarly, Zaglia et al. (2015) examined the scope of optogenetics in assessing the function of Purkinje fibers in a minimally invasive manner in intact hearts because the availability of such a direct, selected, and non-invasive manipulation of the conduction system was very limited at that time. They developed transgenic mouse models expressing ChR2 in Purkinje fibers, under the control of Cre recombinase and cell-specific promoter (Cx-40) (Cx-Cre mouse; Zaglia et al., 2015). They utilized LED source and optical fibers for epicardial illumination of the Purkinje network. They found that electrophysiological responses (e.g., ectopies) were very dependent on the illumination site and such responses were abolished upon ablation of Purkinje fibers, confirming the correct targeting of ChR2 expression (Zaglia et al., 2015). Thus, these studies show the great potential of optogenetics to investigate and interrogate the role of cardiac conduction system of an intact heart in a minimally invasive manner, both during and after development.

### Monitoring Electrophysiological Functions

**Figure 3:**
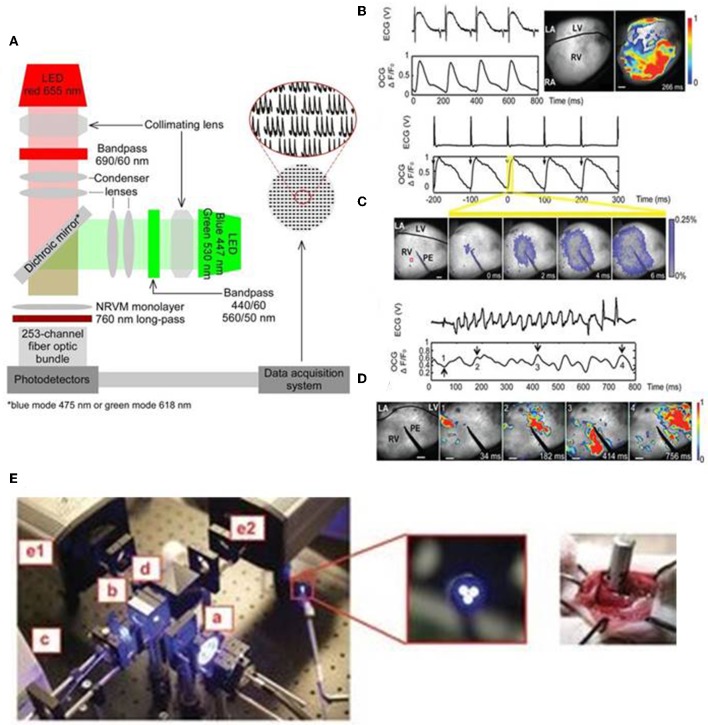
An all optical system by Park et al. includes two LEDs for concurrent stimulation and fluorescence recording from monolayers of ChR2- or eNpHR3.0-expressing neonatal rat ventricular myocyte (NRVM), stained with the voltage-sensitive dye PGH1, appropriate collimating lens, 253-channel fiber optic bundle, photodetectors, and data acquisition system **(A)**. The generation of optical cardiogram (OCG) waveforms in Langendorff-perfused transgenic mouse hearts expressing the optogenetic voltage sensor VSFP2.3, under the control of the cardiomyocyte-selective MHC promoter, using fast camera system (frame rate 500 Hz) **(B–D)**. OCG recordings were synchronous with the ECG recordings during sinus rhythm **(B)**. The OCG and ECG recordings showed 1:1 capture during electric pacing at physiological rate (10 Hz) using point electrode (PE) **(C)**. OCG also showed anisotropic spread of signal over time (**C**, below), reflecting the physiologic nature of signal propagation. Both ECG and OCG reflected abnormal recordings following induction of ventricular arrhythmia (using 12.5-Hz 200-pulse train) and arrhythmic regions can be located using OCG signal propagation images captured at regular time intervals **(D)** (Scale bars: 1 mm). Fiber optic system (**E**, left) also tested to record OCG signals from the intact hearts of transgenic mice, expressing VSFP2.3, in a minimally invasive manner. The fiber optic system consists of light source (a), excitation filter (b), optical fiber bundle (c), emission filter (d), cameras for capturing CFP, and YFP fluorescence signals (e1 and e2) (**E**, middle: magnified image showing tip of fiber bundle; right: image showing direct placement of fiber bundle against the functioning heart *in situ*). Reproduced with permission from Park et al. (2014) and Chang Liao et al. (2015).

Optical mapping of cardiac biological functions, such as transmembrane voltage and calcium changes, provide important insights on the physiological processes during cardiac development, homeostasis and disease (Chang Liao et al., 2015; Xu et al., 2017). Traditional methods of recording such electrophysiological phenomena include: (1) surface electrodes which are limited by their spatial resolution and (2) intracellular methods, such as impaling electrodes and patch clamps, which are invasive, time-consuming, and low throughput (Chang Liao et al., 2015; Xu et al., 2017). Organic dyes, such as fluorescent dyes that label specific intracellular ions (Ca, Mg, Na) and voltage sensitive dyes, provide higher spatial resolution, higher temporal resolution and high content optical imaging not possible with traditional microelectrodes (Chang Liao et al., 2015; Koopman et al., 2017). In addition, integration of optogenetics and such fluorescent imaging allows for control of the functions of multiple cardiac cells and also simultaneous mapping of their biological signals non-invasively (Entcheva, 2013; Park et al., 2014). Park et al. (2014) shows capability to monitor, control and map action potential of neonatal rat ventricular myocytes by expressing ChR2 or eNpHR3.0 and voltage-sensitive dye (PGH1) and through concurrent opsin stimulation and fluorescence excitation of such cells. An example of an optical system utilized by Park et al. is illustrated in Figure 3A. Jia et al. also previously demonstrated all optical system (with additional closed feedback system) for contactless and precise optical actuation, control and mapping of biological signals, such as calcium transients and membrane potential (Jia et al., 2011). Appropriate selection of dyes and optogenetic constructs is crucial in such optogenetic system so that their absorption spectra do not infringe with one other (Jia et al., 2011; Park et al., 2014). However, organic dyes, such as voltage-sensitive dyes, are hindered by their chronic toxicity, inability to perform repeated experiments on the same sample over an extended period of time and the applicability of such dyes for *in vitro* studies only (Chang Liao et al., 2015; Koopman et al., 2017).

Optogenetic sensors, such as GEVIs and GECIs, can address some of the aforementioned limitations with conventional electrodes and organic dyes and provide precise means of probing functions of intact heart cells in a minimally invasive manner (Chang Liao et al., 2015; Koopman et al., 2017; Xu et al., 2017). Specifically, genetically encoded voltage sensors enable direct *in vivo* readout of the voltage signals, such as action potential, of the intact cells (Xu et al., 2017). In addition, when such sensors are integrated with optogenetic actuators they provide all optics system, which enable precise controlling and mapping of the electrical activity of opsin-expressing intact cells and tissues (Park et al., 2014). In addition, cell-specific promoters and sub-cellular specific motifs allow targeted expression to specific cell types and sub-cellular regions, respectively, that provide higher spatial resolution (Koopman et al., 2017). Liao et al. established the first transgenic mouse model expressing the voltage sensor VSFP2.3, under the control of the cardiomyocyte-selective α-MHC promoter (Chang Liao et al., 2015). They reported that transgene expression did not elicit cardiotoxicity but produced highly reliable optical recordings of cardiomyocyte-specific membrane voltage of Langendorff-perfused transgenic mouse hearts and such optical recordings were synchronous with the ECG recordings at both normal and arrhythmic conditions (Figures 3B–D; Chang Liao et al., 2015). The optical system by Liao et al. provides a minimally invasive procedure for optical recordings of cardiomyocyte-specific voltage changes in an intact transgenic mouse heart and the components of the system, which includes light source, excitation filter, optical fiber bundle, emission filter, and camera, are detailed in Figure 3E (Chang Liao et al., 2015). These studies suggest that targeted expression of optogenetic sensors and appropriate optical system provide a valuable tool in monitoring electrophysiological functions of the target cells, such as cardiomyocytes, in a minimally invasive manner in both *in vitro* and *in vivo* settings.

### Manipulating Electrophysiological Functions and Disease Models

**Figure 4:**
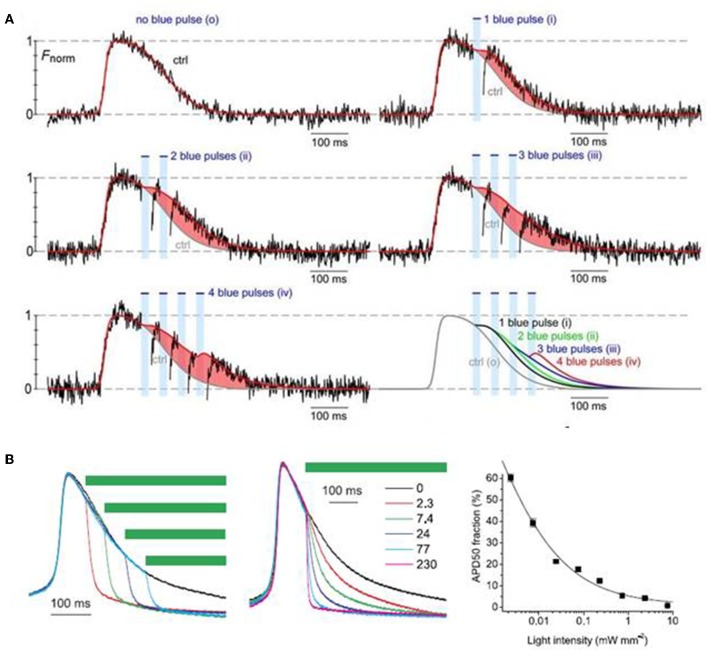
Optogenetic manipulation of action potential duration using light stimuli of an appropriate wavelength. Action potential duration of ChR2-sensitized cardiomyocytes can be prolonged upon application of blue light pulses during the repolarization phase of the cells and the increment in action potential duration is dependent on the number of light pulses **(A)**. On the contrary, action potential of cardiomyocytes expressing GtACRs, an anion channelrhodopsin, can be shorted using green light pulses (510 nm), where the action potential shortening is dependent both on the instance of illumination and light intensity **(B)**. Reproduced with permission from Park et al. (2014) and Govorunova et al. (2016).

Park et al. (2014) examined the feasibility of monitoring and manipulating action potentials (lengthening or shortening duration) of opsin-expressing cardiomyocytes using an all optical system. They demonstrated that action potential duration of ChR2- or eNpH3.0-sensitized monolayer of neonatal rat ventricular myocytes can be reliably modulated, prolonged, or dampened using light stimulus of appropriate wavelength, frequency and intensity (Figure 4; Park et al., 2014). Next, Govorunova et al. (2016) demonstrated an efficient approach for shortening of cardiac action potential by using an anion channelrhodopsin from *Guillardia theta* (GtACRs) which provide selective and higher anion conductance to cause cell inhibition with lower light intensity, when compared to traditional halorhodopsin (NpHR). They found that action potential duration of cultured NRVM was shortened to variable extents by delivering pulsed light (510 nm) at different intervals or of different intensities (e.g., 2.3–230 uW/mm^2^) during the repolarization phase (Figure 4; Govorunova et al., 2016). These results suggest that optogenetics potentially provide therapeutic mechanisms in treating heart diseases arising from altered electrophysiology, such as long QT syndrome. Many lethal arrhythmias are result of pathological changes in action potential duration and very few drugs are capable of addressing such issues without significant side effects (Park et al., 2014; Govorunova et al., 2016; Boyle et al., 2018). Thus, optogenetics can be explored as a novel tool to modulate electrophysiological functions, mimic heart diseases and potentially treat such outcomes.

### Pacing the Heart and Treating Arrhythmias

#### Background

Ischemic heart diseases, cardiomyopathies, channelopathies, myocarditis, genetic abnormalities, and congenital defects may manifest uncoordinated electrophysiological functions in the heart and often result to arrhythmias, which is one of the leading causes of sudden cardiac arrest (Spartalis et al., 2018). Typically, sudden cardiac death accounts for ~50% of all cardiovascular deaths wherein ventricular arrhythmias alone account for ~80% of sudden cardiac deaths occurring in public (Spartalis et al., 2018). Anti-arrhythmic drugs, ion channel blockers, catheter ablation, implantable electronic pacemakers and defibrillators are some of the commonly adopted clinical methods for treating cardiac arrhythmias (Ambrosi and Entcheva, 2014; Van Weerd and Christoffels, 2016; Spartalis et al., 2018).

Electronic pacemaking is considered the first line treatment of bradyarrhythmias and for resynchronization therapies. It causes brief depolarization of a small group of cardiomyocytes which consequently causes whole heart excitation (Bruegmann et al., 2016). Cardioverters and defibrillators, on the other hand, are considered for termination of malignant arrhythmias, such as ventricular tachycardia and ventricular fibrillation, and requires depolarization of a larger mass of cardiomyocytes and larger energy when compared to that needed for heart pacing (Zipes et al., 1975; Ambrosi and Entcheva, 2014; Bruegmann et al., 2016). Although these devices are usually considered safe and reliable, they also possess some inherent limitations (Ambrosi and Entcheva, 2014). Electronic pacemakers require invasive surgical procedure, repeated battery replacements and are prone to device malfunctions and infections (Nussinovitch and Gepstein, 2015). Defibrillators cause non-selective excitation of nerves and muscles and deliver high energy electrical shocks which may result in greater tissue damage, pain and discomfort that often lead to patient intolerance to the procedure and increased mortality (Ambrosi and Entcheva, 2014; Bruegmann et al., 2016). These electrical procedures also lead to irreversible electrochemical reactions which cause release of toxic gases and change in tissue pH (Bruegmann et al., 2010; Nussinovitch and Gepstein, 2015). Unfortunately, patients with cardiac conduction abnormalities may need to be treated frequently for a longer period of their life; hence, application of such electrical therapies may be restricted because of repeated cardioversion shocks, toxic gas release, myocardial tissue damage, pain, and increased mortality associated with the procedure (Boriani et al., 2000; Geller et al., 2003; Bruegmann et al., 2010). Hence, there is an unmet clinical need of a novel procedure that can be applied permanently to treat cardiac conduction abnormalities in a rapid, precise, controlled, less-traumatic, and shock-free manner (Nyns et al., 2019).

#### Developments in the Field

**Figure 5:**
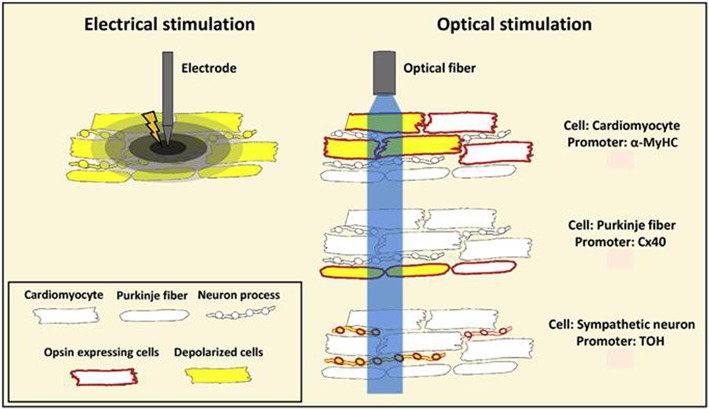
A schematic representation showing benefits of optogenetic stimulation over conventional electrical stimulation. Conventional electrical stimulation results in an unspecific depolarization of myocardial cells, including nerve fibers, within the stimulated tissue volume resulting in pain and tissue injury. Optogenetics provides restricted stimulation to opsin-expressing cells. Listed in the figure are cell-specific promoters Myosin Heavy Chain (MyHC), Connexin 40 (Cx40), and Tyrosine Hydroxylase (TOH) that are used for targeted stimulation of opsin expressing cardiomyocytes, Purkinje fibers and sympathetic neurons, respectively. Reproduced and adapted with permission from Pianca et al. (2017).

Optogenetics hold significant potential as a novel therapeutic procedure to treat conduction abnormalities as it allows light-mediated targeted and controlled stimulation to address issues associated with existing electrical therapies, such as non-selective excitation, electric shocks, unwanted tissue damage, and pain (Figure 5; Ambrosi and Entcheva, 2014). Historically, Bruegmann et al. (2010) first demonstrated that an intact transgenic mouse heart expressing ChR2 can be stimulated rapidly by spatially confined blue light pulses. Specifically, they showed that the heart rate could be entrained by short (1 ms), low-intensity (<10 mW/mm^2^) light pulses delivered to the atria or ventricles and ventricular extrabeats were induced with prolonged ventricular illumination (200 ms; Bruegmann et al., 2010). Thus, this study was the first to show the potential of optogenetics in investigating the mechanisms and conditions associated with cardiac pacing and arrhythmias (Bruegmann et al., 2010).

**Figure 6:**
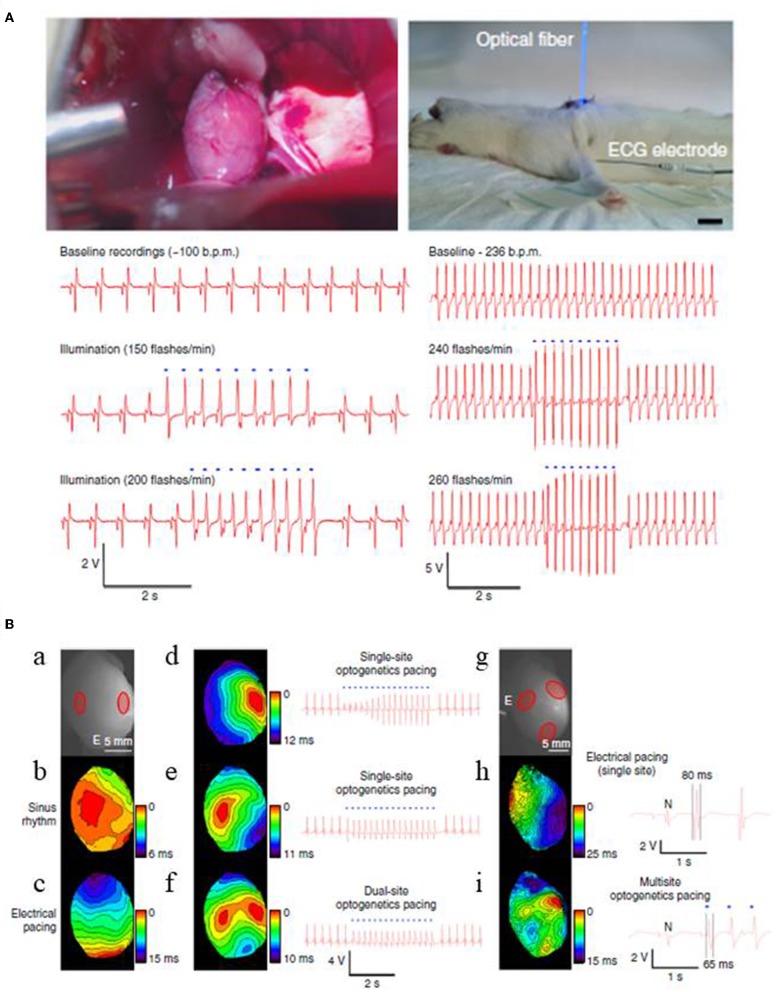
Optogenetic pacing of rat hearts in an open chest (left) and a closed chest (right) configurations **(A)**. Apical expression of ChR2 was obtained via intra-myocardial delivery of transgene-carrying AAV-9 vector in the apex of rat hearts. Two weeks after the transgene delivery photosensitive hearts were illuminated with blue light, using LED-coupled fiber optic. For both configurations, ECG recordings show that heart beating rates increase with the flashing frequency of the light stimulus in a 1:1 ratio. Blue dots in the figure designate blue-light illumination (Scale 1 cm). Cardiac resynchronization therapy using multi-site optogenetic pacing for isolated rat hearts, expressing ChR2 **(B)**. Two red circles mark ChR2 delivery sites for dual site-optogenetic pacing while white lettered “E” designates the placement of electrode during electrode pacing (a), optical maps and ECG recordings during: normal sinus rhythm (b), electrical pacing at the apex (c), single-site optogenetic pacing (d,e), and dual-site optogenetic pacing (f). Three red circles refer ChR2 delivery sites (g), optical maps and ECG recordings during: single-site electrical pacing (h), and multi-site optogenetic pacing (i). Multi-site optogenetic pacing lowered both the left ventricle activation time (14.8 ms, see Optical map) and also the QRS durations (65 ms, see ECG recordings) compared to those values from single-site right ventricular electrical pacing (i.e., 24.5 and 80 ms, respectively). Reproduced with permission from Nussinovitch and Gepstein (2015).

Optogenetics also enables excitation of only a small number of target cells. Thus, it may be useful in advancing current pacemaker technology that uses ventricular pacing, rather than direct, physiological His bundle pacing, due to the physical constraints of pacing wire positioning (Nussinovitch and Gepstein, 2015; Sharma et al., 2015; Boyle et al., 2018). Recent computational and experimental studies show that optogenetics will address such challenges by providing targeted stimulation of opsin-expressing His bundle only, but not of the neighboring cells, and also by lowering the energy requirements associated with the procedure (Boyle et al., 2013, 2018; Zaglia et al., 2015). Another concern with the conventional pacing procedure is that it rely on single site ventricular pacing, an approach that often alters or prolongs total ventricular activation time and reduces ventricular contractile function and further deteriorates systolic performance (Wilkoff et al., 2002). This concern has been clinically addressed using biventricular pacing procedure, an approach termed as cardiac resynchronization therapy (CRT), but the procedure is impeded by the limited number of pacing wires (Nussinovitch and Gepstein, 2015). To address these shortcomings, Nussinovitch and Gepstein (2015) investigated the potential of optogenetics for cardiac pacing and CRT in rat hearts that were injected with a ChR2-AAV-9 vector at one or more specific sites. They found that the heart rate could be entrained by blue light (450 nm) pulses both *in vivo* and *ex vivo* (Figure 6; Nussinovitch and Gepstein, 2015). Further, they found that upon simultaneous photo stimulation of three different opsin expressing sites in the left ventricle, the total ventricular activation time was significantly lowered (14.8 ms) when compared to single-site right ventricular electrical pacing (24.8 ms) (Figure 6; Nussinovitch and Gepstein, 2015).

**Figure 7:**
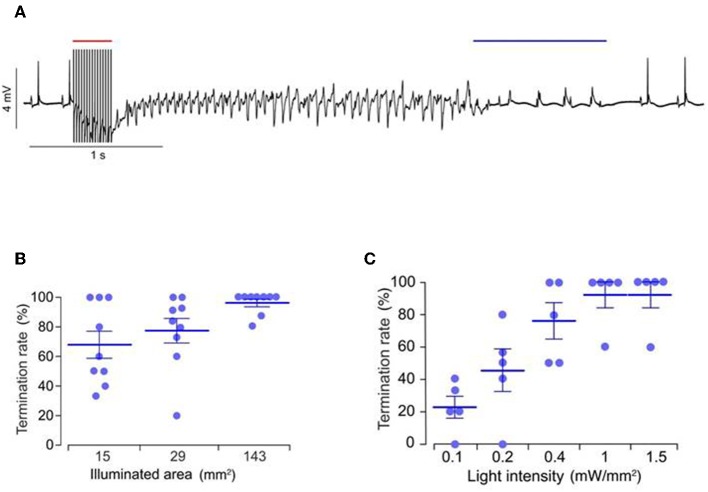
Representative ECG signals of an explanted mice heart, expressing ChR2 **(A)**. Ventricular tachycardia was induced via electrical stimulation (red bar, 50 Hz) and was terminated via epicardial illumination with blue light (blue bar, 470 nm, 1 s, 0.4 mW/mm^2^, 143 mm^2^). Role of illumination parameters, such as duration **(B)** and area **(C)**, on arrhythmia termination rate are presented. Reproduced with permission from Bruegmann et al. (2016).

DC shock effectively terminates cardiac fibrillation, but the unspecific delivery of high electrical shocks used for defibrillation can cause greater tissue damage (Karagueuzian and Chen, 2001). Thus, there is a need for less damaging and low energy defibrillation methods. Bruegmann et al. (2016) for the first time examined the potential of optogenetic defibrillation in treating ventricular tachycardia of intact hearts. They experimentally demonstrated that anteroseptal epicardial illumination of the ChR2 transgenic mouse hearts, using single light pulse (470 nm wavelength, 0.4 mW/mm^2^ intensity, 143 mm^2^ illumination area, and 1 s exposure duration), terminated electrically induced sustained ventricular tachycardia (success rate ~85%) (Figure 7; Bruegmann et al., 2016). They also determined the illumination parameters for ventricular tachycardia termination, such as an effective illumination duration should capture total ventricular tachycardia duration (80 ms) while light intensity should be increased (>0.4 mW/mm^2^) when the illumination area is reduced (<143 mm^2^) (Figure 7; Bruegmann et al., 2016). Concomitant *in silico* work showed that the same approach in the human heart would require light pulses of longer wavelength (669 nm) and higher intensity (>10 mW/mm^2^; Bruegmann et al., 2016).

Similarly, Burton et al. (2015) and Crocini et al. (2016) investigated possibilities in managing the heterogeneity of the conduction system, such as the characteristic re-entrant conduction waves, for efficient restoration of cardiac rhythm. In specific, Crocini et al. induced and characterized ventricular tachycardia in ChR2-expressing mouse hearts, loaded with the voltage sensitive dye di-4-ANBDQPQ, and employed mechanistic-based custom stimulation in an all optical system to possibly inhibit re-entrant waves by refractories of ChR-2 stimulated cardiomyocytes for optogenetic defibrillation (Crocini et al., 2016). They compared the efficiencies of a single point, a single barrier and a triple barrier stimulation patterns were compared in terminating arrhythmias, and found that the triple barrier was the most effective in interrupting ventricular tachyarrythmias (Crocini et al., 2016). They also found that although the success rates of triple barrier and whole heart stimulation were comparable, the total irradiance energy associated with each procedure were significantly different (0.25 and 1 mJ, respectively), showing the therapeutic potential of optogenetic-based cardioversion procedures (Crocini et al., 2016). Zaglia et al. (2015) demonstrated the utility of optogenetics for identifying specific heart regions, areas, cell numbers, and cell types (His-Purkinje vs. working myocytes) that contribute to arrythmogenesis following myocardial ischemia. These studies, along with an excellent review paper by Pianca et al. (2017), demonstrate the therapeutic potential of optogenetics in treating arrhythmias.

**Figure 8:**
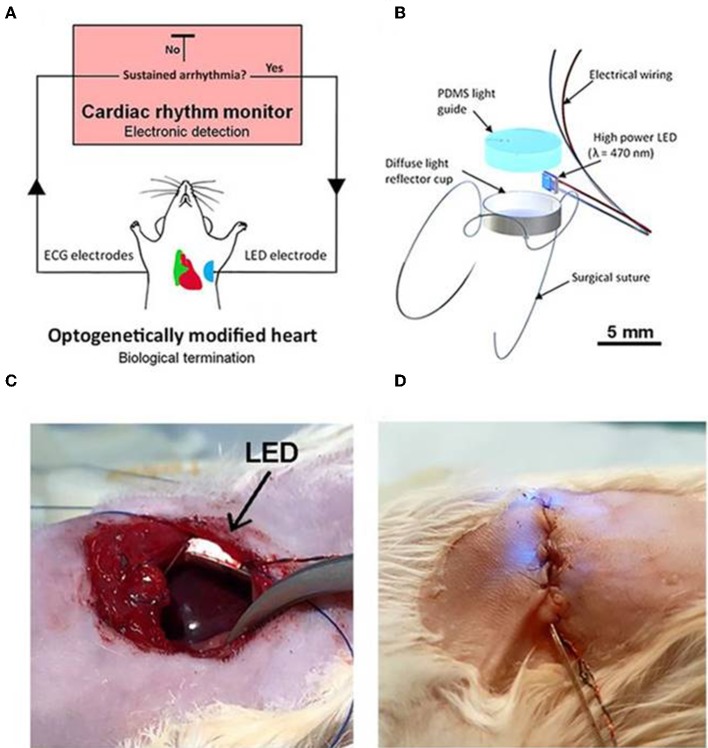
An automated hybrid bioelectric system for the restoration of sinus rhythm during atrial fibrillation. The autogenous bioelectric system comprises of an opsin expressing right atrium (green), a cardiac rhythm monitor, and ECG electrodes, where the ECG signal is fed as input to the cardiac monitor **(A)**. Abnormalities in ECG recordings, such as during atrial fibrillation, causes an output signal that triggers ON the implantable LED device (500 ms) which activates ReaChR in the atrium; the activation of the latter terminates fibrillation and restores normal sinus rhythm. Detail on the LED assembly **(B)**, experimental details on LED implantation procedures, before **(C)** and after the chest closure **(D)**, respectively, are illustrated. LED is held firmly on the thoracic wall and on the top of the right atrium without any physical contact to the heart **(C)**. Electrical wires for pacing and turning on LED are shown in **(D)**. Reproduced with permission from Nyns et al. (2019).

Recently Nyns et al. (2019) also investigated the therapeutic potential of an automated hybrid bioelectric system for safe, precise, repeated, shock free, and on-demand termination of atrial fibrillation. They first found that arrhythmias that were induced in ReaChR- expressing-isolated and intact rat hearts, were terminated via spatially confined epicardial illumination of the right atrium (20 mm^2^) using single light pulses (470 nm, 3.5 mW/mm^2^) of 100 ms and 1,000 ms durations (Nyns et al., 2019). Next, they implanted a LED system, controlled by cardiac rhythm monitor system and triggered by the onset of atrial fibrillation, in the rat's thoracic cavities (Nyns et al., 2019). They demonstrated that 96% of induced atrial fibrillation episodes were efficiently terminated upon LED activation (470 nm, 3.5 mW/mm^2^, 500 ms); a detailed schematic and experimental set up of the system is provided in Figure 8. These results suggest that integration of optogenetic and electrical systems would provide a novel mechanism for autogenous detection and termination of arrhythmias. Such a technique could provide an electrical, shock-free, and anti-fibrillatory therapy which is in high demands in younger patients suffering with recurrent episodes of atrial fibrillation (Nyns et al., 2019).

### Pre-clinical Drug Development

Cardiotoxicity is one of the primary reasons for drug withdrawal. In the United States, it contributes to ~20% of withdrawal at each phase of drug development and post-market surveillance process (Piccini et al., 2009; Klimas et al., 2016). Current assays of evaluating electrophysiology, as a measure of cardiotoxicity testing, are low throughput, rely on the physical contact, are difficult to scale up and automate (Ambrosi et al., 2014; Entcheva and Bub, 2016; Boyle et al., 2018). Thus, efficient high throughput screening tools for detection of drug-induced cardiotoxicity at early phase of drug development process would significantly reduce the cost of the process (Piccini et al., 2009). Klimas et al. (2016) demonstrated the first automated all optical system for dynamic cardiac electrophysiology measurement, named OptoDyCE, for rapid assessment of cardiotoxicity measure of drug candidates. They tested with ChR2-expressing generic cardiomyocytes and iPSC-derived cardiomyocytes using voltage- and calcium-sensitive dyes to enable high-content and high-throughput acquisition of drug-induced electrophysiological changes in the action potential and associated calcium transients (Klimas et al., 2016). In addition, Entcheva et al. also suggested that such all optical high-throughput system allows precise probing and monitoring of cell electrophysiology within 3D constructs or within an intact organs, such as heart (Entcheva and Bub, 2016). These studies suggest that optogenetics, together with all optical system, hold potential in pre-clinical evaluation of cardiotoxicity during drug development through rapid, contactless and automated procedures for monitoring cellular functions in a high throughput fashion.

### Cell-Cell Coupling

**Figure 9:**
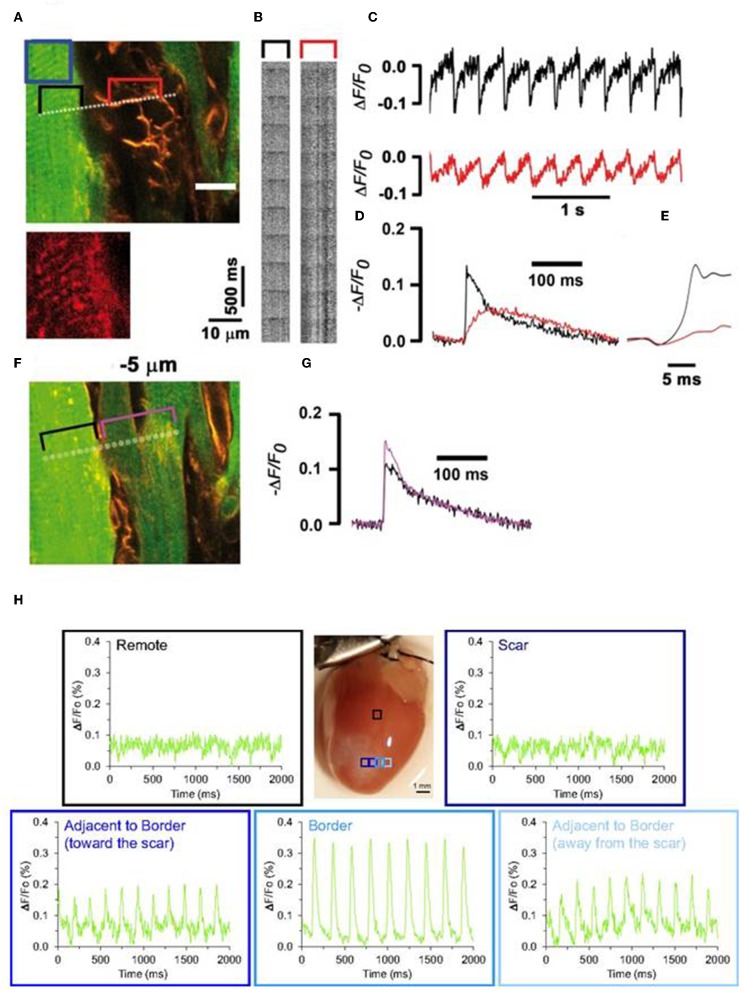
Entrainment of the membrane potential of non-cardiomyocytes by ventricular cardiomyocytes in an infarct border of a Langendorff-perfused mouse heart **(A–G)**. Mouse hearts were genetically engineered to express cardiomyocyte-specific EGFP to identify cardiomyocytes from non-cardiomyocytes. Prior two-photon laser scanning microscopy (TPLSM), harvested transgenic hearts were loaded with voltage-sensitive fluorescence dye (ANNINE-6plus). Isolated hearts were electrically paced (3 Hz) at remote regions and fluorescence data in the myocytes and adjacent non-myocytes were recorded using multiple line-scan acquisition system across the tissue. A line crossing over cardiomyocytes and non-cardiomyocytes (**A**, dotted line) was repeatedly scanned to acquire line scan fluorescence F(x,t) **(B)**. Traces showing ΔF/Fo **(C)** were generated from line-scan data; black trace denotes signal from cardiomyocytes (from black bracket in **A**) while red trace denotes signals from adjacent non-myocytes (from red bracket in **B**). Magnified section **(F)** and successive readings **(G)**. Transmembrane potential in both these cell types are in phase (1:1 ratio) suggesting the heterocellular electric coupling between myofibroblasts and cardiomyocytes in the infarct border. Reproduced with permission from Rubart et al. (2017). Evidence of such heterocellular coupling between cardiomyocytes and non-cardiomyocytes in the scar border is also evident from a study by Quinn et al. **(H)**. Intact transgenic mouse hearts where non-cardiomyocytes, selectively made to express VSFP2.3, showed enhanced electrical activity, as observed by YFP signal in the scar border. Reproduced with permission from Quinn et al. (2016).

Optogenetics hold great potential in investigating heterocellular coupling between cardiomyocytes and non-cardiomyocytes, both on healthy and infarcted hearts (Yu et al., 2017). Investigators have shown the role of heterocellular coupling between scar tissue (myofibroblasts) and un-injured cardiomyocytes *in situ* following infarction and such couplings are suspected to induce adverse outcomes, such as arrhythmias (Miragoli et al., 2006; Jacquemet and Henriquez, 2007; Rubart et al., 2017). Quinn et al. (2016) utilized an optogenetic protein, VSFP2.3, to report for the first time on the existence of heterocellular electrotronic coupling between cardiomyocytes and non-cardiomyocytes in intact transgenic mouse hearts, following cryoinjury. They provided targeted expression of VSFP2.3 in cardiac cells of mouse hearts using Cre-lox recombination and cell-type specific promoters and reported that signals from non-myocyte, expressing VSFP2.3, at the border of injury were similar to that of action potential of cardiomyocytes. These results suggest that optogenetics provide a valuable tool in determining heterocellular electrotronic coupling between cardiomyocytes and non-cardiomyocytes in an intact transgenic mouse heart at healed borders (Figure 9; Quinn et al., 2016); such results are in agreement with our recent observations from chronically infarcted mouse hearts that were loaded with a voltage-sensitive fluorescent dye ANNINE-6plus (Rubart et al., 2017). In brief, Langendorff-perfused mouse heart, induced with myocardial infarction injury and stained with ANNINE-6plus, were electrically paced (3 Hz) at remote regions and the fluorescence data, indicating the transmembrane voltages, in the myocytes and adjacent non-myocytes were recorded using multiple line-scan acquisition data (Figure 9; Rubart et al., 2017). We also observed that transmembrane potential in non-myocytes fluctuates in phase with myocytes (1:1 ratio), which infers that non-myocytes were electrically coupled with cardiomyocytes in infarct border via heterocellular coupling (Figure 9; Rubart et al., 2017). However, some of the potential advantages of using optogenetic proteins (e.g., VSFP2.3), instead of organic dyes for such applications, would be their non-toxicity, non-cardiotoxicity, and feasibility of performing repeated *in situ* observations on target cells for a longer period of time (Chang Liao et al., 2015; Koopman et al., 2017).

Enhanced coupling between cardiomyocytes and non-cardiomyocytes after myocardial injury is mediated by phenotypic changes in the fibroblasts, which results in an increased connexin expression and also in an emergence of nanotubes-like structures between the cell types (Quinn et al., 2016; Yu et al., 2017). Determination of heterocellular coupling is also useful in assessing the electrophysiological maturity of stem-cell derived cardiomyocytes (e.g., iPSC-CM) following transplantation, since electrical coupling between transplanted cells and native cardiomyocytes may indicate the conductivity and the functionality of the graft in the recipient tissue (Boyle et al., 2018). Thus, optogenetics can greatly aid in the *in situ* understanding of heterocellular coupling in the normal, diseased, or tissue-engineered cardiac tissues.

### Modulation of Cell Signaling

Optogenetics is not only limited to the use of light-sensitive ion channels and pumps but the field is also being driven toward other light-sensitive proteins, such as G-protein modulating opsins (Table 1; Ferenczi et al., 2019). In cardiac research, both natural and engineered versions of these opsins are being investigated to activate cell signaling with high temporal and spatial precision (Ferenczi et al., 2019). A recent study by Makowka et al. (2019) has reported the utility of jellyfish opsin (JellyOp) to activate G_s_ signaling in cardiomyocyte embryoid bodies and also in the intact mice hearts. Upon stimulation of JellyOp embryoid bodies with blue light (470 nm, 2.9 mW mm^−2^, 5 min), cAMP levels were increased for up to 646% of the baseline levels (Makowka et al., 2019). In addition, beating rates of the embryoid bodies and of the intact transgenic mice hearts were increased upon optogenetic stimulation (Makowka et al., 2019). Similar effects were observed in JellyOp control groups, stimulated with pharmacologic agents; however, light termination render significantly faster recovery to the baseline states in the transgenic groups when compared to wash out of isoprenaline in the control groups (Makowka et al., 2019). In another recent study, Repina et al. investigated the optogenetic control of canonical Wnt signaling (OptoWnt) via optical stimulation of hESC expressing photoreceptor Cryptochrome 2, fused to Wnt co-receptor LRP6 (Repina et al., 2019). They examined that OptoWnt caused reduced expression of pluripotency markers but produced concomitant increment in mesendoderm differentiation (Repina et al., 2019). Interestingly, they also reported that optogenetics enabled activation of Wnt signaling in subpopulation of cells which also showed cell self-organization in 2D and 3D cultures, mimicking the phases of human gastrulation (Repina et al., 2019). Similarly, another earlier work by Beiert et al. (2014) showed that illumination of cardiomyocytes expressing melanopsin (a light-activated G_q_ coupled receptor found in the photosensitive ganglion cells of the retina) (Berson et al., 2002; Qiu et al., 2005) increased their beating rates and induced a local pacemaker activity within the illuminated regions (Beiert et al., 2014). These studies show that optogenetics also hold great potential in deciphering the temporal and spatial role of cell signaling in cardiac research and morphogenesis.

## Future Directions

Despite these advances, cardiac optogenetics is still in a nascent stage for clinical translation (Gruber et al., 2018). One key approach to move closer to human application would be in scaling up the system to larger animal models, such as pig, which would better mimic human heart anatomy (Boyle et al., 2018). In specific, both the genetic engineering tools (for appropriate opsin variant types, opsin characteristics, methods, and levels of opsin expression) and also the optical systems (for appropriate source and mode of light delivery, pulse characteristics, illumination area, duration, and intensity) should be optimally determined in these larger animal models (Nyns et al., 2019). Next, emphasis should also be given to the *in silico* approaches that provide appealing tools in modeling different systems, such as heterogeneity in opsin expression, light absorption, illumination pattern, and in scaling up the system to meet clinically relevant sizes and also in predicting the outcomes accordingly (Boyle et al., 2013; Karathanos et al., 2016). On the other hand, since most of the present day observations are obtained from unconscious and immobilized animal models, another strategy to advance toward translational medicine would be to develop technologies feasible on freely moving and conscious experimental animals; such models would best mimic the native physiological states (Gruber et al., 2018). Furthermore, integration of recent developments on biocompatible integumentary membrane and micro LED array can expedite development of optogenetic membrane that can contract along the myocardial surface and provide optical stimulation at multiple locations simultaneously; such technology is particularly appealing for optogenetic defibrillation method that requires stimulation of a larger myocardial area (Boyle et al., 2015).

One of the key considerations for clinical application of cardiac optogenetics is on the safe, stable, and uniform opsin expression in human heart (Pianca et al., 2017). Concerns of toxicity and immunogenicity may arise with opsin expression in cardiac applications that requires high opsin levels for robust photocurrents (Boyle et al., 2015; Gruber et al., 2018). Besides, safe and appropriate vehicle types and administration routes must be well-established for intended applications (Ambrosi et al., 2014). In this regard, although many novel technologies are emerging to address such needs, they are yet to be validated in appropriate animal models. For instance, tandem cell unit has only been validated in *in vitro* settings because of possible issues of cell engraftment and host-graft coupling for *in vivo* applications (Pianca et al., 2017; Gruber et al., 2018). Next, possible concerns of non-uniform opsin expression (which may render heterogeneous cell depolarization and induce substrate for arrhythmias), ectopic opsin expression and immune concerns with host-specific antibodies to viral vectors, etc. need to be explored in depth in clinically relevant larger animal models (Pianca et al., 2017). Thus, clinically acceptable vehicle, delivery method, safe level, and retention period of opsin expression, all should be carefully determined. Fortunately, highly efficient and safer transgene knock in strategies such as CRISPR/Cas9 system are being highly investigated recently. Applications of such advanced genetic engineering methods would greatly reduce barriers of non-uniform or ectopic opsin expression with conventional strategies for cardiovascular research and regenerative medicine.

Another concern with the clinical translation of optogenetics is the limitations on light penetration due to highly dense and light-scattering cardiac tissues, specifically with applications of blue light for the excitation of ChR2 opsins (Entcheva, 2013; Boyle et al., 2015). Potential alternatives to such issues are applications of: (1) red-shifted optogenetic proteins to increase light penetration depth, (2) ultrathin LEDs to maintain beam shape and minimize light scattering, (3) bioluminescence techniques to fuse opsin with luciferase and eliminate the need of external light illumination, (4) opsins co-expressed with sonoluminescent or x-ray inducible nanophosphors to release visible light *in situ* and reduce light attenuation, and (5) up-conversion nanoparticles to convert highly tissue penetrable near infrared waves to visible light (Entcheva, 2013; Boyle et al., 2015, 2018; Jiang et al., 2017). Another area that demands thorough investigation is on the energy requirements of optogenetic-based therapies for cardiovascular applications. The overall energy for an optogenetic-based therapy is influenced by light penetration, optical properties of the tissues, level, pattern, and functionality of opsin expression, and the physiological properties of the host cells/tissues, making it difficult to predict the energy requirements for *in vivo* applications (Entcheva, 2013). Despite these considerations, encouraging *in vitro* and *in vivo* studies have provided proof-of concept for the translational potential of cardiac optogenetics. In addition, emerging scope of optogenetics in modulating the dynamic roles of signaling moieties needed during crucial biological functions, such as cell signaling, differentiation and migration are greatly expanding the dimensions of *in vitro* and *in vivo* research. Future investigations on such emerging scopes would greatly enhance the clinical success of the field for cardiovascular regenerative medicine.

## Author Contributions

JJ and WZ wrote the manuscript. MR revised the manuscript.

### Conflict of Interest

The authors declare that the research was conducted in the absence of any commercial or financial relationships that could be construed as a potential conflict of interest.
